# Prevalence and risk factors of asymptomatic bacteriuria among children living with HIV in Lagos, Nigeria

**DOI:** 10.11604/pamj.2018.31.181.16028

**Published:** 2018-11-14

**Authors:** Adeseye Michael Akinsete, Chinyere Ezeaka

**Affiliations:** 1 Department of Pediatrics, College of Medicine, University of Lagos/Lagos University Teaching Hospital, Idi-Araba, Lagos, Nigeria

**Keywords:** Asymptomatic bacteriuria, HIV/AIDS, risk factors

## Abstract

**Introduction:**

HIV/AIDS has gradually become a chronic disorder following the success of combination chemotherapy. As a result of the persisting immune deficiency, certain risk factors predispose affected individuals to infections. The aim of the study was to determine the prevalence and identify risk factors of asymptomatic bacteriuria among HIV infected children.

**Methods:**

this was a case control study conducted at the Lagos University Teaching Hospital from July 2010 to June 2011.Eighty-five children living with HIV were consecutively selected from the HIV clinic of the Lagos University Teaching Hospital and compared with 85 age and sex matched HIV negative controls for the occurrence of asymptomatic bacteriuria. Mid-stream urine samples were obtained from the participants and the samples were analyzed for microscopy, culture and sensitivity. Demographic and clinical data was obtained from the caregivers and clinical notes respectively. Data were analyzed utilizing SPSS version 17.

**Results:**

the prevalence of asymptomatic bacteriuria was 24.7% among children living with HIV and 8.2% among un-infected children (p value 0.004). The stage of the disease, CD4 count, sex as well as age were risk factors for asymptomatic bacteriuria among children living with HIV.

**Conclusion:**

asymptomatic bacteriuria is a prevalent problem among children living with HIV infection and urinary screening should be routine in the work up of febrile children living with HIV.

## Introduction

Urinary tract infection (UTI) is a common cause of morbidity with childhood epidemiology varying with several factors [1-3]. It is also a common cause of renal disease in childhood [1, 2]. In the United States, cumulative incidence of UTI before the age of six years is 3% to 7% in girls and 1% to 2% in boys [1]. In Nigeria, the prevalence differs across the different geo-political zones with reported rates of 11.96% in a semi-urban setting in the South-West, 2.7% in an urban setting in the South East and 13.7% in Northern Nigeria [4-6]. Asymptomatic bacteriuria is defined as the presence of 105 colony forming units per millimeter of the same organism/s in two consecutive urine samples of individuals without clinical symptoms [7]. Asymptomatic bacteriuria among healthy children may be beneficial but among children with sickle cell anemia, diabetes, pregnant adolescents and those with depressed immunity, it may predispose to recurrent UTI and possible renal scarring [8]. The Human Immunodeficiency Virus (HIV) and Acquired Immune Deficiency Syndrome (AIDS) is a major cause of infant and childhood morbidity and mortality affecting an estimated two million children worldwide with sixty seven percent of the affected children residing in sub-Saharan Africa [9]. Presently, the use of highly active antiretroviral therapy (HAART) has succeeded in improving clinical outcomes for children with HIV/AIDS thereby prolonging their life [10, 11]. The main effect of HIV is its preference for T-lymphocytes and natural killer cells which renders them unable to phagocytize and allowing low virulence pathogens to cause infections [12]. This study hypothesized that asymptomatic bacteriuria will be commoner among children infected with HIV and also documented the risk factors of asymptomatic bacteriuria.

## Methods

A case-control study was conducted at the Lagos University Teaching Hospital (LUTH), Idi-Araba from July 2010 to June 2011. Two categories of children were enrolled in the study, 85 HIV infected children and 85 age and sex matched controls. Permission was obtained from the care-givers and assent from the children. Ethical approval was obtained from the Health, Research and Ethics Committee of LUTH. Participants with fever, signs of urinary tract infection and prior use of antibiotics within 14 days of the enrolment were excluded. The diagnosis of HIV infection was by documentary evidence of ELISA®, confirmed by Western Blot®. The controls were age and sex matched apparently healthy HIV negative subjects. Following counselling, the HIV screening test was carried out using ELISA. A post-test counselling was done before results were given in private and strict confidence. None of the children who were enlisted as controls tested positive to HIV. Mid-stream urine samples were collected from the children into sterile wide mouthed containers. The caregivers were carefully instructed about urine collection and same printed on a card for the caregivers. The urethral orifice was cleaned with sterile water for the male children before voiding. Subsequently, the children were allowed to void. The caregivers obtained the urine from the mid-portion of the urine stream after the children had started voiding. For children who were old enough, this procedure was explained to them. Two consecutive samples were sent to the laboratory within the hour [13]. For the female children, the peri-urethral area and perineum were cleaned by the caregivers with sterile water in a forward to backward motion and this was repeated once. The girls then voided and the caregiver collected the mid-stream urine with a wide mouthed sterile container. Two consecutive samples were sent to the laboratory within the hour [13]. The urine samples were examined in the laboratory under the microscope for casts, erythrocytes, leucocytes and sediments after centrifuging.

The standard loop technique was adopted to place five microliters of the urine in Blood and MacConkey agar media respectively. The media were incubated at 37°c for 18-24 hours using a Gallenkamp® incubator. Those without growth were re-incubated for another 24 hours. Samples showing ≥10^5^ bacterial colonies per milliliter of urine are considered significant bacteriuria. The bacterial isolates were identified using the Analytical Profile Index (API) 20E identification system according to the manufacturer's manual. Susceptibility testing was done on the identified bacteria using the modified Kirby Bauer technique [14] on Muller Hinton agar (OXOID). The following antibiotic discs were used: 1. Ampicillin (AMP), Oxacillin (OXA), Cefuroxime (CRX), Co-Amoxiclav (AUG), Cefotaxime (CTX), Gentamycin (GEN), Ciprofloxacin (CIP), Co-trimoxazole (COT), Ceftazidime (CAZ), Nitrofurantoin (NIT) The sensitivity plates were incubated aerobically for 18 hours and the zone of inhibition noted [15]. Data were inputted, validated and analyzed using SPSS version 17. Descriptive and inferential statistics were utilized in the analysis. Continuous variables were expressed as means and standard deviation. Proportions and percentages were calculated for categorical variables. The means of continuous variables were compared using the student's t-test while categorical variables were compared using Pearson's Chi square and Fisher's exact test. A p value less than 0.05 was accepted as statistically significant (two-tailed analysis).

## Results

A total of 170 children participated in the study with a mean age of 8.68 ± 2.96 years. There were slightly more males than females with a ratio of 1.2:1. Most of the participants were in the middle to lower socio-economic classes (Table 1). The prevalence of asymptomatic bacteriuria was 24.7% among children living with HIV and 8.2% among uninfected children (Table 2). Among the different age groups studied, asymptomatic bacteriuria was significantly more among adolescent than any other age group. It was also more prevalent among the younger age groups when compared with un-infected children (Table 3). Asymptomatic bacteriuria was also significantly more prevalent among the female participants (Table 3). It was noticed that the prevalence was more among those with severe disease (Table 4). Interestingly, socio-economic status was only a factor among the un-infected study participants (Table 5).

**Table 1:** socio-demographic characteristics of respondents

Characteristics	HIV positive n = 85	HIV negative n = 85	*x*^2^	p value
**Age (years)**				
5 – 9	53 (62.4%)	53 (62.4%)		1
10 – 14	29 (34.1%)	29 (34.1%)	-	
≥ 15	3 (3.5%)	3 (3.5%)		
Mean age	8.68 (2.96%)	8.72 (3.07%)	-	0.07
**Gender**				
Female	37 (43.5%)	41 (47.8%)	0.11	0.74
Male	48 (56.5%)	44 (52.2%)		
**Mother’s education**				
No formal education	2 (2.4%)	3 (2.9%)		0.01^*^
Primary	16 (18.8%)	9 (10.1%)		
Secondary	37 (43.5%)	25 (30.4%)	-	
Tertiary	23 (27.1%)	48 (56.5%)		
Unknown (orphan)	7 (8.2%)	0 (0%)		
**Socio-economic class**				
Upper (i and ii)	21 (24.7%)	45 (52.9%)	21.63	0.01^*^
Middle (iii)	15 (17.6%)	20 (23.5%)		
Lower (iv and v)	49 (57.7%)	20 (23.5%)		

* p is significant

**Table 2:** prevalence of asymptomatic bacteriuria in HIV positive and negative children

HIV positive n = 85(100%)	HIV negative n = 85 (100%)	
21 (24.71%)	7 (8.24%)	Bacteriuria
64 (75.29%)	78 (91.76%)	No bacteriuria

*x^2^* = 8.38, p = 0.004, OR=3.7

**Table 3:** prevalence of asymptomatic bacteriuria by age and gender

Age(years)	Cases n = 85(100%)		Controls n= 85(100%)	
	Bacteriuria 21 (24.7%)	No bacteriuria 64 (75.3%)	Bacteriuria 7 (8.2%)	No bacteriuria 78 (91.8%)
**Age**				
5 – 9	9 (10.6%)	44 (51.8%)	4 (4.7%)	49 (57.6%)
10 – 14	10 (11.8%)	19 (22.4%)	3 (3.5%)	26 (30.7%)
≥15	2 (2.3%)	1 (1.1%)	0 (0.0%)	3 (3.5%)
p = 0.01^*^ (p* is significant)				
**Gender**				
Male	6 (7.0%)	42 (49.4%)	2 (2.4%)	42 (49.4%)
Female	15 (17.7%)	22 (25.9%)	5 (5.8%)	36 (42.4%)

*x^2^* = 21.22, p = 0.00^*^ (p* is significant)

**Table 4:** prevalence of asymptomatic bacteriuria by WHO clinical stages

Clinical stage	Bacteriuria n = 21(24.7%)	No bacteriuria n = 64(75.3%)	*x^2^*	fisher’s exact p value
I	1 (1.2%)	3 (3.5%)		0.02^*^
II	3 (3.5%)	30 (35.4%)		
III	12 (14.1%)	25 (29.4%)		
IV	5 (5.9%)	6 (7.0%)		

**Table 5:** prevalence of asymptomatic bacteriuria by socioeconomic class

Socio-economic class	HIV negative n= 7	HIV positive n=21
**i & ii**	1 (14.3%)	4 (19.0%)
**iii**	0 (0.0%)	5 (23.8%)
**iv & v**	6 (85.7 %)	12 (57.2%)
	7 (100.0)	21 (100.0)

*x^2^* = 0.96, Fischer’s Exact p = 0.62

## Discussion

The prevalence of asymptomatic bacteriuria was found to be expectedly higher among participants with HIV/AIDS. This finding is comparable to a report from Benin [16] that reviewed the prevalence rate among children. Furthermore, reports among adolescents and adults documented that the risk of asymptomatic bacteriuria was more in HIV affected individuals [17, 18]. The risk of asymptomatic bacteriuria was found to be higher with increasing age. This is consistent with the report from Benin [16] that documented that the rate increased with age. The prevalence steadily increased from the age group 10-14 years till the age group 15 years and above, where over two-thirds of the HIV infected participants had asymptomatic bacteriuria. This is similar to the report from Ile-Ife among children and adolescents where the prevalence of asymptomatic bacteriuria was found to increase with the age of un-infected subjects [3]. The un-infected cohort in this study did not support the assertion of increased asymptomatic bacteriuria rates among un-infected adolescents. The number of adolescents in this study were however too small to draw any inference. The increased asymptomatic bacteriuria rates among adolescents has been attributed to sexual activity which has been found to be common among this age group [19].

This study also found that the prevalence of asymptomatic bacteriuria was significantly higher among female participants corroborating what has been documented in published literature [19] and the reasons commonly adduced for female susceptibility to asymptomatic bacteriuria are the relatively shorter length of the urethra, the proximity of the urethra to the anal opening and the absence of a prostatic like fluid in the female which is thought to be bacteriostatic [20, 21]. It was also observed that the rate of asymptomatic bacteriuria increased significantly with the severity of the disease with the rates in WHO stages 3 and 4 twice more than those in stages 1 and 2 and this was consistent with the report of Hoepelman *et al.* in Netherland in 1992 and Klapacynska *et al*. in Poland in 2018 who reported increased rates of UTI among subjects with lower CD4 counts [22, 23]. The authors of the Dutch study [22] reported that no respondent with CD4 count greater than 500cells/mm^3^ had UTI and this was corroborated by Evans *et al.* [24] who reported from the United Kingdom that UTI was commoner amongst HIV infected subjects with CD4 count less than 200cells/mm^3^. While the Polish study [23] was retrospective in nature, the Dutch study [22] was prospective but only included males who were in same sex relationships. However, both studies were in adult populations unlike ours in a strictly pediatric population. Surprisingly, the socio-economic status of the family as well as the mother's educational status were not risk factors for asymptomatic bacteriuria among children infected with HIV. This is similar to what was reported from Benin [16]. The reverse was however noted among the controls where asymptomatic bacteriuria was more prevalent in mothers who had less than secondary education and also those who were from the lower socio-economic classes. We assumed that mothers of HIV infected children will have better health seeking and health maintaining practices because of the regularity of hospital/clinic visits.

## Conclusion

This study has shown that the risk of asymptomatic bacteriuria is significantly higher in children living with HIV than among un-infected children. The stage of the disease, the more immunologically depressed children and female adolescents are more at risk of asymptomatic bacteriuria. Routine yearly screening for asymptomatic bacteriuria should be recommended for optimal management of these children.

### What is known about this topic

- HIV infection has been reported as a risk factor for asymptomatic bacteriuria in children and adults.

### What this study adds

- Low CD4 counts, age and sex are risk factors for asymptomatic bacteriuria in children living with HIV;
- Socio-economic status was not a significant risk factor for asymptomatic bacteriuria among children living with HIV.

## Competing interests

The authors declare no competing interests.

## References

[1] Ross JH, Kay R (1999). Paediatric urinary tract infection and reflux. Am Fam Physician.

[2] Abdulrahman MB, Amir KI, Shamran IO (1992). Urinary tract infection in children is still a mismanaged problem. Emirates Med J.

[3] Zorc JJ, Kiddoo DA, Shaw KN (2005). Diagnosing and management of paediatric urinary tract infection. Clin Micr Rev.

[4] Aiyegoro OA, Igbinosa OO, Ogunwonyi IN (2007). Incidence of urinary tract infections among children and adolescent in Ile-Ife, Nigeria. Afr Microbiol Res.

[5] Chukwu BF, Okafor HU, Ikefuna AN (2011). Socio-demographic factors associated with asymptomatic bacteriuria in children with sickle cell anaemia in a tertiary health facility in South-Eastern Nigeria. Nigerian Medical Journal.

[6] Rabasa AI, Gofama MM (2009). Urinary tract infection in febrile children in Maiduguri, North-Eastern Nigeria. Niger J Clin Pract.

[7] Kass EH (2002). Asymptomatic infection of the urinary tract. J Urol.

[8] Nicolle LE (2003). Asymtomatic Bacteriuria, when to screen and treat. Infect Dis Clin North Am.

[9] Joint United Nations Programme on HIV/AIDS (UNAIDS) Report on the global AIDS pandemic, 2016.

[10] Gibb DM, Duong T, Tookey PA (2003). National Study of HIV in Pregnancy and Childhood Collaborative HIV Paediatric Study: Decline in mortality, AIDS, and hospital admissions in perinatally HIV-1 infected children in the United Kingdom and Ireland. BMJ.

[11] McConnell MS, Byers RH, Frederick T (2005). Trends in antiretroviral therapy use and survival rates for a large cohort of HIV infected children and adolescents in the United States, 1989-2001. J Acquir Immune Defic Syndr.

[12] Weller I, Roit I, Brostoff J, Male D (2006). Secondary Immunodeficiency. Immunology.

[13] Washington W Koneman's Atlas and Textbook of Diagnostic Microbiology.

[14] Collee JG, Fraser AG, Marmion BP (1996). Practical Medical Microbiology.

[15] Cheesbrough M (1933-1940). Biochemical test to identify bacteria; antimicrobial susceptibility testing, In: District Laboratory Practice in Tropical countries.

[16] Iduoriyekewen NJ, Sadoh WE, Sadoh AE (2012). Aymptomatic Bacteriuria in HIV infected Nigerian children. JMBR.

[17] Omoregie R, Eghafona NO (2009). Urinary tract infection among asymptomatic HIV patients in Benin-city. Nigeria Br J Biomed Sc.

[18] Inyang-Etoh PC, Udofia GC, Alaibe AA (2009). Asymptomatic bacteriuria in patients on antiretroviral drug treatment in Calabar. J Med Sci.

[19] Hooton TM, Scholes D, Hughes JP (1996). A prospective study of risk factors for symptomatic urinary tract infection in young women. N Engl J Med.

[20] Kennedy T (2003). Rudolph's Pediatrics.

[21] Sharma S (1997). Current understanding of pathogenic mechanisms in UTI. Ann Natl Acad Med Sci.

[22] Hoepelman A, Van Buren M, Van De Broek J (1992). Bacteriuria in men infected with Human Immunodeficiency Virus-1 is related to their immune status (CD4+ cell count). AIDS.

[23] Klapacynska AS, Matlosz B, Bednarska A (2018). Factors associated with urinary tract infections among HIV-1 infected patients. PLOS one.

[24] Evans JK, Mcowan A, Hillman FJ (1995). Incidence of symptomatic urinary tract infections in HIV seropositive patients and the use of cotrimoxazole as prophylaxis against pneumocystis carinii pneumonia. Genitourin Med.

